# Case Report: Radiological Features of Sclerosing Epithelioid Fibrosarcoma in the Right Fibula

**DOI:** 10.3389/fonc.2020.603127

**Published:** 2020-11-18

**Authors:** Qiaoling Ding, Xiaotong Shao, Xiaocao Liu, Yanbiao Fu, Fengbo Huang, Chao Wang

**Affiliations:** ^1^ Department of Radiology, The Second Affiliated Hospital, Zhejiang University School of Medicine, Hangzhou, China; ^2^ Department of Pathology, The Second Affiliated Hospital, Zhejiang University School of Medicine, Hangzhou, China

**Keywords:** bone tumor, fibula, sclerosing epithelioid fibrosarcoma, contrast-enhanced computed tomography, magnetic resonance imaging

## Abstract

**Background:**

Sclerosing epithelioid fibrosarcoma (SEF) is an extremely rare, aggressive malignant subtype of fibrosarcoma. Only dozens of cases of primary SEF in the bone have been reported so far, without case involving fibula reported in literature to date. Herein we report the first case of primary SEF in the right fibula in a 19-year-old man. In this case report, we firstly give a comprehensive description of fibula SEF, including its complete clinical course and radiological findings.

**Case Presentation:**

A 19-year-old man presented with a half-year history of soreness in the right lower leg. Contrast-enhanced computed tomography (CE-CT) and magnetic resonance imaging (MRI) of the right lower leg were performed. Based on the radiological examinations, a diagnosis of malignant tumor arising in the fibular diaphysis was made. Final diagnosis of primary SEF in the right fibula was confirmed by histopathological and immunohistochemical examinations after surgical resection. The patient had no signs of recurrence or metastasis during a 24-month follow-up.

**Conclusion:**

We report an exceedingly rare case of primary SEF in the right fibula and its radiological features with CE-CT and MRI.

## Introduction

Sclerosing epithelioid fibrosarcoma (SEF) is an extremely rare, aggressive and malignant subtype of fibrosarcoma described initially by Meis-Kindblom and grouped under low-grade fibrosarcoma in 1995, which is characterized by small and cytologically bland epithelioid cells arrayed in cords, nests or sheets embedded in a dense collagenous extracellular matrix (1). It has been reported to have relatively high incidence of local recurrence (>50% of cases), metastasis (40–80% of cases) and tumor-related mortality (2, 3). SEF typically involves the extremities or the torso (2), followed by the abdominal viscera (4, 5) and head and neck areas (6–8). Primary SEF in the bone was first reported in 2001 (9). And it is exceedingly rare which may cause diagnostic pitfalls radiologically. Only dozens of cases of primary SEF in the bone have been reported so far (10–14), without case involving fibula reported in literature to date. Herein we report the first case of primary SEF in the right fibula in a 19-year-old man. In this case report, we firstly give a comprehensive description of fibula SEF, including its complete clinical course and radiological findings.

## Case Presentation

### Clinical History

A 19-year-old man presented with soreness in right leg for over half a year, which was tolerable without limb numbness. The symptom was not severe or aggravated by movement. He had no history of recent trauma or injuries. Then, he was admitted to the Department of Osteology of the Second Affiliated Hospital of Zhejiang University School of Medicine due to a soft tissue mass in the right fibula found by plain X-ray. Coagulation blood tests revealed that prothrombin time was 14.8 s (normal range: 12–14 s). Routine blood laboratory tests, including full blood count, erythrocyte sedimentation rate (ESR), electrolyte panels, renal and liver function test were normal, except 49.8% neutrophils (normal range: 50–70%) and 43.5% lymphocytes (normal range: 20–40%). And routine fecal and urine laboratory tests including a fecal occult blood test indicated normal findings. Routine serum tumor biomarkers were all within normal ranges, including carcinoembryonic antigen (2.7 ng/mL), carbohydrate antigen 125 (CA125, 4.3U/ml), carbohydrate antigen 199 (CA199, 2.0 U/ml), alpha fetoprotein serum (2.0 ng/mL), and prostate-specific antigen (0.245 ng/mL).

### Radiological Examinations

**Figure 1:**
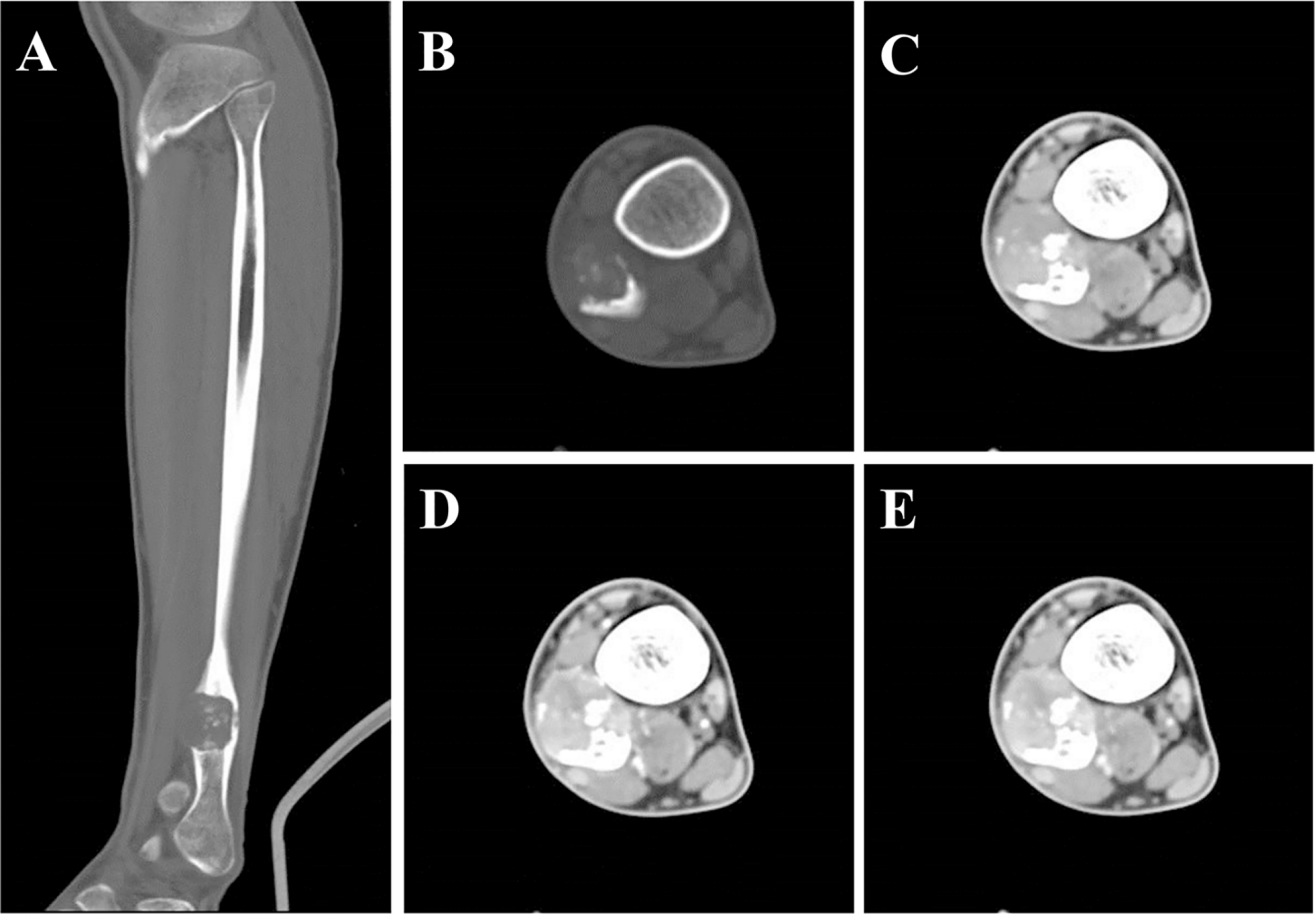
CT showed a lesion of bone destruction in the right distal fibula. Sagittal **(A)** and axial **(B)** CT of bony window showed a centrally osteolytic lesion with an expansive growth pattern, scattered punctate bony sclerosis inside and a local, slight periosteal reaction. Axial CT of soft tissue window images demonstrate a well-demarcated soft tissue mass displayed uneven density in unenhanced phase (**C**, CT value = 93 HU) and mild enhancement in the arterial phase (**D**, CT value = 113 HU) and moderate heterogeneous enhancement in the portal phase (**E**, CT value = 123 HU).

**Figure 2:**
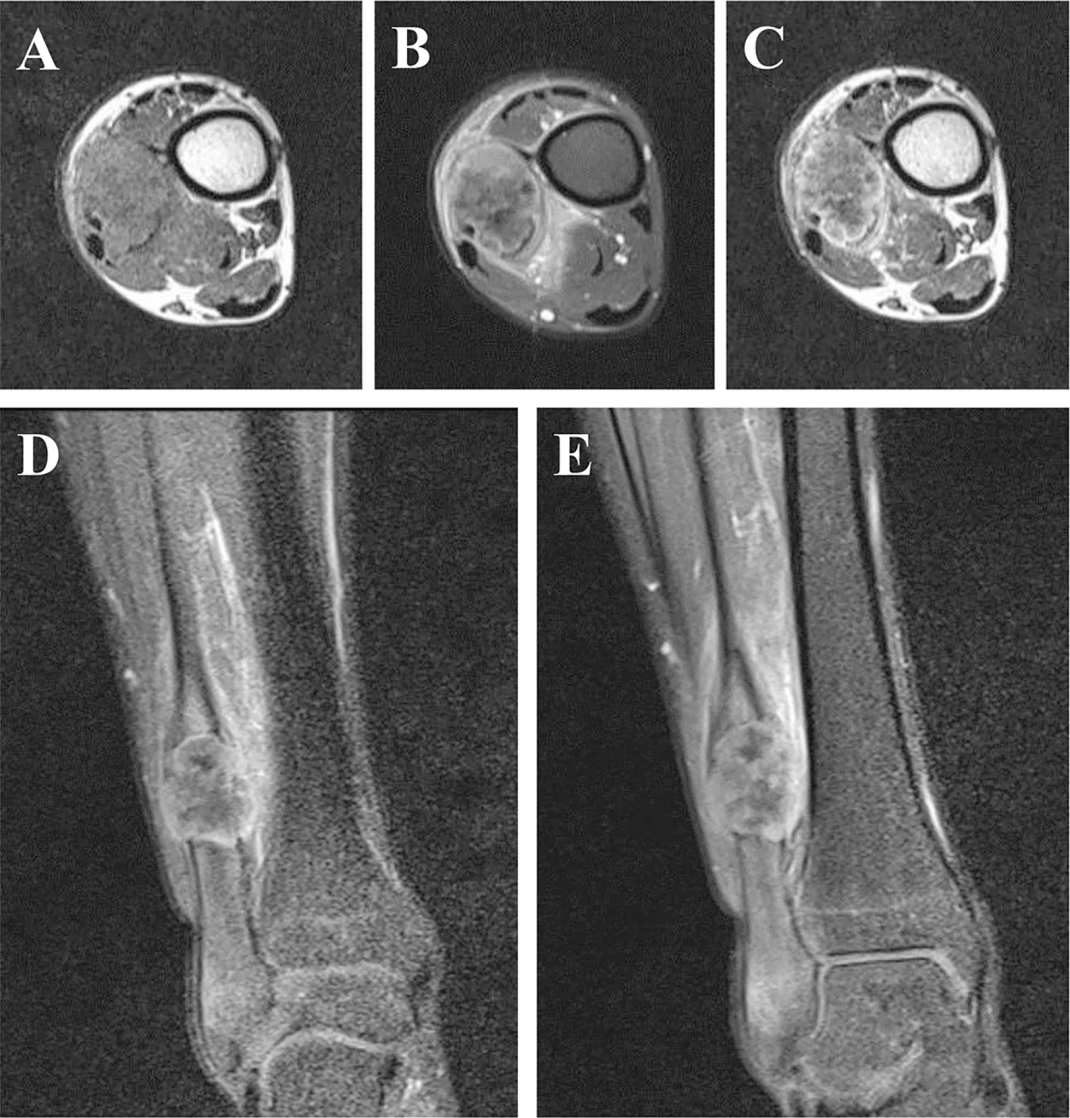
Axial T1-weighted image without contrast enhancement. **(A)**, T2-weighted image **(B)** and the arterial phase of T1-weighted image **(C)**. The arterial **(D)** and the venous **(E)** phase of coronal T1-weighted images. MRI revealed a focal, hypo- and isointense mixed mass on T1-weighted image **(A)**, and hypo- and hyperintense mixed mass on T2-weighted image **(B)**. There were no signs of necrosis, hemorrhage, or cyst formation within the mass. However, adjacent soft tissue edema and swelling was seen. Gadolinium-enhanced T1-weighted images revealed obvious perilesional enhancement, particularly in the region adjacent to the normal tissue **(C–E)**.

Plain X-ray revealed an irregular, low-density and well-demarcated region of bone destruction in the right distal fibula with scattered patches of slightly high density inside and slight periosteal reaction. Slight swelling change of the adjacent soft tissue was also observed. Computed tomography (CT) images of bony window showed centrally expansile osteolytic changes with scattered punctate bony sclerosis inside (**Figures 1A, B**). Axial CT images demonstrated a soft tissue mass with heterogeneous density (**Figure 1C**) and mild enhancement in the arterial phase (**Figure 1D**) and moderate enhancement in the venous phase (**Figure 1E**). There were no signs of fibular artery, venous, or small saphenous venous invasion. Magnetic resonance imaging (MRI) revealed a focal, 2.5cm × 3.0 cm × 3.2 cm, hypo- and isointense mixed mass on T1-weighted images (T1WI) (**Figure 2A**), and hypo- and hyperintense mixed mass on T2-weighted images (T2WI) (**Figure 2B**). There were no signs of necrosis, hemorrhage, or cyst formation within the mass. However, adjacent soft tissue edema and swelling was seen. Gadolinium-enhanced T1WI revealed obvious perilesional enhancement, particularly in the region adjacent to the normal tissue (**Figures 2C–E**). The MRI report suggested an osseous malignant tumor in the right distal fibula.

### Surgical Findings and Pathological Examination Results

**Figure 3:**
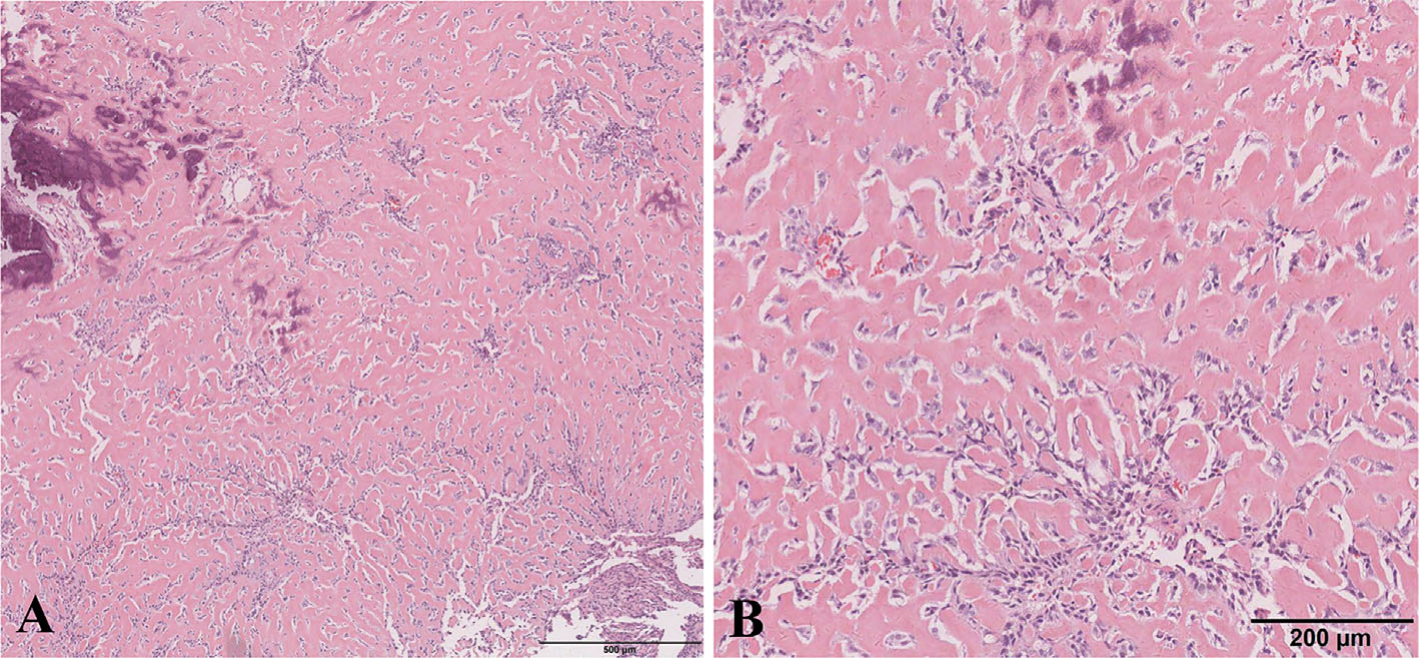
Histological images. **(A)** The tumor consisted of extensive areas of a densely sclerotic hyaline matrix with fibroblastic epithelioid cells arranged in cords, nests, or sheets inside accompanied by partial calcification (hematoxylin and eosin staining; original magnification: × 4). **(B)** Cells were arranged in nests or clusters (hematoxylin and eosin staining; original magnification: × 10).

Based on the radiological diagnosis, on March 5th, 2018, tumor resection of the right distal fibula and external fixation with autogenous fibular graft and reconstruction were performed. The tumor was well-demarcated, and located in the right distal fibula 6 cm from the right ankle without infiltrating the surrounding blood vessels and nerves. No visible metastatic nodules were found surrounding the tumor. Histological staining showed that fibroblastic epithelioid cells were ovoid and arranged in nests, cords, or sheets within a collagen-rich extracellular matrix (**Figure 3**). Immunohistochemical staining showed the tumor cells were positive for CD31 (weak positive), ERG, Fli-1, and Myoglobin, and negative for CD34, S-100, smooth muscle actin (SMA), Desmin, CK (AE1/AE3), CAM5.2, MyoD1, and Myogenin. The Ki-67 proliferation index was low (5%) in tumor cells. A diagnosis of primary SEF in the fibula was finally determined.

### Post-Operative Course

**Figure 4:**
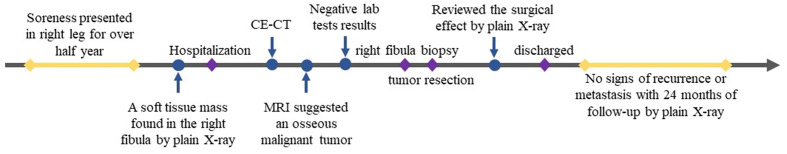
The timeline of diagnosis and treatment.

After 24 months of follow-up, the patient had no signs of recurrence or metastasis. The timeline of diagnosis and treatment was shown in **Figure 4**.

## Discussion

SEF is an extremely rare variant of fibrosarcoma, which is considered as a low-grade fibrosarcoma (1, 9). However, SEF is an aggressive tumor with high rates of recurrence, metastasis and mortality (1, 9). It mostly occurs in the soft tissues. Only a handful of cases have been reported to occur primarily in bone (9, 10, 14, 15), without case involving fibula reported in literature to date. To the best of our knowledge, this case is the first reported case of primary SEF in the fibula. Primary osseous SEF mainly affects patients with a mean age of 45 years and has no gender predilection (10). Nevertheless, this patient was a 19-year-old young man.

We reviewed all the cases of primary SEF in long bones in the reported literatures (**Supplementary Table**). Of these 11 cases, there were 5 males and 6 females with an age range of 8–73 years. There were 6 cases in the femur, 2 cases in the ulna, 1 case in the humerus, 1 case in the tibia and 1 case in the fibula. Of these cases, only 3 cases showed CT and/or MR images (10, 14, 16). In this case report, we firstly give a comprehensive radiological description of fibula SEF, including CT and MR findings. Primary osseous SEF usually manifested as a well-demarcated, heterogeneous, osteolytic mass with bone destruction on CT (10, 13). The mass typically presented a sharp border without obvious periosteal reaction (10, 13). In the present case, the osteolytic lesion also presented a well-demarcated sharp border with slight periosteal reaction. In addition, irregular areas of low signal intensity was found within the mass on T2WI (**Figure 2B**) and this area showed no enhancement on gadolinium-enhanced T1WI (**Figures 2C–E**), which may present the area of decreased cellularity and dense fibrous tissue or collagen deposition (17). Obvious perilesional enhancement of the tumor adjacent tissue region was found on gadolinium-enhanced T1WI (**Figures 2C–E**), which may be associated with cellular and vascular proliferation, peritumoral desmoplastic reaction and inflammatory cell infiltration (18). Moreover, adjacent soft tissue edema and swelling surrounding the tumor were found in this case. These radiological features may be indicative of a malignant tumor. Therefore, in the present case, imaging findings successfully suggested a malignant tumor containing fibrous tissue.

Radiological features, especially MRI features, may provide biological and histopathological information relating to SEF. However, the radiological differential diagnosis of primary osseous SEF in the fibula is challenging because of its rarity. Because primary osseous SEF occurs in the bone marrow and presents prominent osteoid-like sclerosis and occasional ossification, SEF is at risk to be misdiagnosed as osteosarcoma before surgery. Nevertheless, osteosarcoma usually show a marked periosteal reaction on plain X-ray and CT, and has no area of low signal intensity within the mass on T2WI of MRI.

Surgical resection remains the most effective treatment for SEF (19). It remains unclear about the effectiveness of systemic adjuvant therapy in improving the progression of SEF (20, 21). Because of its aggressive behavior, SEF usually shows a poor prognosis. Therefore, regular radiological follow-up is needed. As far as this 19-year-old young man was concerned, he was advised to maintain regular follow-up after treatment. After 24-month follow-up, the present case had no signs of recurrence or metastasis.

In summary, this case is the first reported case of primary SEF in the fibula. It is difficult to make an accurate diagnosis before surgery due to its rarity. Radiological features, especially MRI features, may provide some biological and histopathological information for preoperative diagnosis. However, it is noted that primary osseous SEF should receive diagnostic consideration for lesion that exhibits a slowly enlarging mass, fibrous tissue inside, and obvious perilesional enhancement.

## Data Availability Statement

The original contributions presented in the study are included in the article/**Supplementary Material**. Further inquiries can be directed to the corresponding author.

## Ethics Statement

The studies involving human participants were reviewed and approved by the Second Affiliated Hospital of Zhejiang University School of Medicine Ethics Committee. The patients/participants provided their written informed consent to participate in this study. Written informed consent was obtained from the individual(s) for the publication of any potentially identifiable images or data included in this article.

## Author Contributions

CW performed the data acquisition. CW and QD performed the radiological images analysis. YF and FH performed the histological images analysis. CW, QD, XS, and XL performed the manuscript preparation. All authors contributed to the article and approved the submitted version.

## Conflict of Interest

The authors declare that the research was conducted in the absence of any commercial or financial relationships that could be construed as a potential conflict of interest.
