# Supplementary material for: Case Report: Radiological Features of Sclerosing Epithelioid Fibrosarcoma in the Right Fibula
Source: Front Oncol. 2020 Nov 18;10:603127. doi: 10.3389/fonc.2020.603127 (PMC7708320; doi:10.3389/fonc.2020.603127)
Supplement: Supplementary file 1 [file Table_1.docx]

Supplementary Table. A summary of clinical and radiological features of long bone SEF.

| Case # | Age (years) | Sex | Location | Maximum diameter (cm) | CT | MRI | Recurrence (R)/ Metastases (M) | Follow-up | Ref. |
| --- | --- | --- | --- | --- | --- | --- | --- | --- | --- |
| 1 | 23 | F | Femur | 5.7 | ND | Yes | M lung | AWD, 52 Mo | [10] |
| 2 | 8 | M | Femur | ND | ND | ND | M skull | AWD, 24 Mo |  |
| 3 | 51 | F | Femur | 5.5 | ND | ND | ND | NED, 60 Mo |  |
| 4 | 58 | M | Femur | 5.2 | ND | ND | M (12 Mo) lung | AWD | [14] |
| 5 | 51 | F | Femur | 9.6 | Yes | Yes | M (1 Mo) sacrum | AWD |  |
| 6 | 73 | F | Ulna | 4 | ND | ND | No | NED |  |
| 7 | 53 | M | Femur | 2.8 | ND | ND | No | NED |  |
| 8 | 25 | M | Humerus | 11.5 | ND | ND | M (19 Mo) Kidney | AWD |  |
| 9 | 31 | F | Ulna (parosteal) | 3 | ND | ND | No | NED |  |
| 10 | 16 | F | Tibia | 4 | ND | Yes | No | NED,22 Mo | [16] |
| 11 | 18 | M | Fibula | 4 | Yes | Yes | No | NED, 24 Mo | Present case |
| M: male; F: female; R: local recurrence; Mo: months; ND: no data; DOD: died of disease; AWD: alive with disease; NED: no evidence of disease. | | | | | | | | | |
